# Visual impairment is associated with physical and mental comorbidities in older adults: a cross-sectional study

**DOI:** 10.1186/s12916-014-0181-7

**Published:** 2014-10-17

**Authors:** Helen Court, Gary McLean, Bruce Guthrie, Stewart W Mercer, Daniel J Smith

**Affiliations:** Institute of Health and Wellbeing, University of Glasgow, Mental Health and Wellbeing Research Group, 1st floor Administration Building, Gartnavel Royal Hospital, 1055 Great Western Road, Glasgow, G12 0XH Scotland UK; General Practice & Primary Care, 1 Horslethill Road, Glasgow, G12 9LX Scotland UK; Primary Care Medicine, Quality, Safety and Informatics Research Group, University of Dundee, Dundee, UK

**Keywords:** Visual impairment, Comorbidity, Primary care

## Abstract

**Background:**

Visual impairment is common in older people and the presence of additional health conditions can compromise health and rehabilitation outcomes. A small number of studies have suggested that comorbities are common in visual impairment; however, those studies have relied on self-report and have assessed a relatively limited number of comorbid conditions.

**Methods:**

We conducted a cross-sectional analysis of a dataset of 291,169 registered patients (65-years-old and over) within 314 primary care practices in Scotland, UK. Visual impairment was identified using Read Code ever recorded for blindness and/or low vision (within electronic medical records). Prevalence, odds ratios (from prevalence rates standardised by stratifying individuals by age groups (65 to 69 years; 70 to 74; 75 to 79; 80 to 84; and 85 and over), gender and deprivation quintiles) and 95% confidence intervals (95% CI) of 37 individual chronic physical/mental health conditions and total number of conditions were calculated and compared for those with visual impairment to those without.

**Results:**

Twenty seven of the 29 physical health conditions and all eight mental health conditions were significantly more likely to be recorded for individuals with visual impairment compared to individuals without visual impairment, after standardising for age, gender and social deprivation. Individuals with visual impairment were also significantly more likely to have more comorbidities (for example, five or more conditions (odds ratio (OR) 2.05 95% CI 1.94 to 2.18)).

**Conclusions:**

Patients aged 65 years and older with visual impairment have a broad range of physical and mental health comorbidities compared to those of the same age without visual impairment, and are more likely to have multiple comorbidities. This has important implications for clinical practice and for the future design of integrated services to meet the complex needs of patients with visual impairment, for example, embedding depression and hearing screening within eye care services.

**Electronic supplementary material:**

The online version of this article (doi:10.1186/s12916-014-0181-7) contains supplementary material, which is available to authorized users.

## Background

The prevalence of visual impairment increases rapidly with age. It is estimated that more than one in ten of the older UK population suffer from significant visual impairment, which rises to one in three of those more than 90-years old [1]. Visual impairment impacts on every part of a person’s life. It is associated with falls [2-5], reduced capacity to carry out everyday activities [6], the need for residential care [7], and is one of the strongest risk factors for functional status decline in community-living people [8]. However, because older people often have multiple health problems [9], many of these individuals may also have additional health conditions which further compromise health and rehabilitation outcomes, including reduced quality of life, disability, increased healthcare costs, increased inpatient admissions and higher death rates [10-15].

Knowledge about comorbidity in visual impairment is sparse, with only a limited number of studies that describe the nature and extent of comorbidities. Crews used US national survey data to examine the prevalence of nine conditions in older people with visual impairment and effects upon functioning [16]. All nine conditions (breathing problems, depression, diabetes, heart problems, hearing impairment, hypertension, joint problems, lower back pain and stroke) were more common in people with visual impairment and were associated with increased difficulties in walking and climbing steps, shopping and socialising [14]. Van Nispen *et al*. also reported an observational study of 296 visually impaired patients and identified that only 25% had no comorbidities and that comorbidity was associated with decreased levels of health-related quality of life [17]. However, although these studies identify that comorbidities are common for people with visual impairment, they are limited by self-report measures and by a relatively limited number of comorbid conditions assessed.

Understanding the nature and extent of comorbidity in this group will help to inform decisions about how medical practice and healthcare services can be improved for individuals with visual impairment, for example, with respect to integration with other healthcare specialities. In this study, we take visual impairment as an index condition and describe the rates and patterns of comorbidity of 29 chronic physical health conditions and eight mental health conditions. To our knowledge, this is the largest single study of visual impairment and comorbidity to date.

## Methods

We used data from the Primary Care Clinical Informatics Unit at the University of Aberdeen for all 1,751,841 registered patients who were alive and permanently registered with 314 Scottish general practices on 31 March 2007. Data on the presence of 32 common chronic physical health conditions (including visual impairment) and 8 mental health conditions were extracted (listed in Additional file 1). The included patients are representative of the whole Scottish population in terms of age, sex and socioeconomic deprivation [18] and a more detailed explanation of the dataset is available elsewhere [9]. GPs do not routinely record detailed quantitative data about sight such as visual acuity, but will code what they consider to be significant morbidity using Read Codes which are the morbidity coding system used in all UK primary care medical records. The set of Read Codes we used was one created by NHS Scotland Information Services Division to define visual impairment for the purposes of analysing an NHS Scotland general practice morbidity recording national dataset (Practice Team Information) (a full list of the Read Codes used is provided in Appendix 1) [19].

Socioeconomic deprivation was measured using the Carstairs deprivation score divided into quintiles (from most affluent to most deprived). The Carstairs score is based on postcode of residence and is widely used in healthcare research as a measure of socioeconomic status [20]. We restricted our analyses to those 65 years old and older, in order to focus on age-related visual impairment. The sample was sub-divided into the following age groups for analysis: 65 to 69 years; 70 to 74; 75 to 79; 80 to 84; and 85 and older.

Age-gender specific rates were generated by the five age groups defined above and by deprivation quintiles for men and women. To control for differences between the two populations in age, gender and deprivation levels we generated standardised prevalence rates by age group (65 to 69 years; 70 to 74; 75 to 79; 80 to 84; and 85 and older), gender and deprivation quintile using the direct method. These age-gender-deprivation standardised rates were then used to calculate odds ratios (ORs) and 95% confidence intervals (95% CI) for those with visual impairment compared to those without visual impairment (controls) for the prevalence of 29 physical conditions (two conditions were excluded: glaucoma since it is a purely ocular condition, and viral hepatitis because only one person with visual impairment had this condition) and eight mental health conditions (depression, alcohol misuse, ‘other psychoactive substance abuse’, learning disability, anorexia/bulimia, ‘anxiety and other neurotic, stress-related and somatoform disorders’, ‘schizophrenia and related conditions’, and dementia), as well as by the number of overall conditions and the number of physical and mental health conditions.

We used t tests to analyse differences for mean age and deprivation score and one-way analysis of variance for differences across age groups and deprivation quintiles. For all statistical analyses, a *P*-value less than 0.05 was considered statistically significant. All analyses were performed in Stata version 13. The NHS Grampian Research Ethics Service approved the anonymous use of these data for research purposes.

## Results

### Demographics

We identified 5,348 (1.8% of the sample) patients with a Read code for visual impairment (Table 1). Women were over-represented in the visual impairment group compared to controls (63.2% versus 57.1% for controls; *P* <0.001). Individuals with recorded visual impairment tended to be older (mean age 80.9 years versus 74.9 years for controls; *P* <0.001), with 37.1% 80 years old or older compared to 12.1% of controls and were also more likely to be socially deprived (visual impairment Carstairs score 0.08 versus controls −0.35; *P* <0.001) although differences between quintiles were small.

### Comorbidities: visual impairment versus controls

Overall 95.0% of individuals with visual impairment had at least one other condition compared to 84.5% of controls (*P* <0.001) (Table 2). Whilst controls were more likely to have one (visually impaired 9.9% versus controls 19.7%; OR 0.55 95% CI (0.50 to 0.60)) or two (visually impaired 14.7% versus controls 19.9%; OR 0.72 95% CI (0.67 to 0.78)) conditions, those in the visual impairment group were significantly more likely to have four conditions (visually impaired 15.4% versus controls 11.7%; OR 1.17 95% CI 1.08 to 1.26) and five or more conditions (visually impaired 37.4% versus controls 17.8%; OR 2.05 95% CI 1.94 to 2.18) (Table 2) (even after standardising for age, sex and social deprivation).

**Table 1:** **Age, gender and deprivation status, visual impairment versus controls**

**Variable**	**Visual impairment**	**No visual impairment**
Total (%)	5,348 (1.8%)	285,821 (98.2%)
Gender (% male)	1,968 (36.8%)	122,745 (42.9%)
Mean age (sd)	80.9 (7.3)	74.9 (8.4)
Deprivation mean (sd)	0.08 (3.3)	-0.35 (3.2)
Age group (years)	**Number (%)**	**Number (%)**
65 to 69	626 (11.7)	82,976 (29.0)
70 to 74	739 (13.8)	70,939 (24.8)
75 to 79	890 (16.6)	57,311 (20.1)
80 to 84	1,111 (20.7)	40,008 (14.0)
85 and older	1,982 (37.1)	34,587 (12.1)
Deprivation quintile	**Number (%)**	**Number (%)**
Least deprived	827 (15.5)	54,281 (18.9)
2	1,132 (21.2)	66,523 (23.3)
3	1,334 (24.9)	65,759 (23.0)
4	977 (18.3)	52,774 (18.5)
Most deprived	1,078 (20.2)	46,484 (16.3)

All difference significant at *P* <0.001. sd, standard deviation.

**Table 2:** **Prevalence and odds ratio for number and type of comorbidities (standardised by age, gender and deprivation score)**

**Total number of morbidities**	**Visual impairment Number (%)**	**No visual impairment Number (%)**	**Odds ratio (95% CI)**
			**(standardised by age, gender and deprivation)**
None	269 (5.0)	41,540 (14.5)	0.43 (0.38 to 0.49)
One	534 (9.9)	56,166 (19.7)	0.55 (0.50 to 0.60)
Two	784 (14.7)	56,927 (19.9)	0.72 (0.67 to 0.78)
Three	935 (17.5)	46,758 (16.4)	1.02 (0.94 to 1.09)
Four	825 (15.4)	33,565 (11.7)	1.17 (1.08 to 1.26)
Five or more	2,001 (37.4)	50,865 (17.8)	2.05 (1.94 to 2.18)
**Total number of physical conditions**			
None	339 (16.4)	46,761 (16.4)	0.39 (0.34 to 0.44)
One	681 (12.7)	63,010 (22.1)	0.59 (0.55 to 0.64)
Two	992 (18.6)	61,917 (21.7)	0.84 (0.78 to 0.90)
Three	1,047 (19.6)	47,356 (16.6)	1.12 (1.04 to 1.19)
Four	840 (15.7)	31,054 (10.9)	1.28 (1.19 to 1.35)
Five or more	1,449 (27.1)	35,723 (12.5)	1.99 (1.87 to 2.12)
**Total number of mental health conditions**			
None	3,242 (60.6)	217,579 (76.1)	0.63 (0.59 to 0.67)
One	1,303 (24.4)	46,993 (16.4)	1.32 (1.23 to 1.41)
Two	606 (11.3)	17,036 (6.0)	1.53 (1.40 to 1.67)
Three or more	197 (3.7)	4,213 (1.5)	1.76 (1.51 to 2.04)

All difference significant at *P* <0.001.

A similar finding was found when restricting analysis only to physical health comorbidities, but differences were greater for mental health comorbidity. Members of the visual impairment group were less likely to have no recorded mental health condition compared to controls (visually impaired 60.6% versus controls 76.1%; OR 0.63, 95% CI 0.59 to 0.67). Furthermore, 15% of the visual impairment group had two or more mental health conditions compared to only 7.5% of controls (*P* <0.001).

### Physical health conditions: visual impairment versus controls

For the visual impairment group, 27 out of 29 physical conditions were significantly more prevalent relative to controls, with only bronchiectasis and migraine showing no significant differences (Table 3). The largest differences after standardisation for age, sex and deprivation were for multiple sclerosis (0R 3.31, 95% CI 2.43 to 4.51) and diabetes (OR 2.76, 95% CI 2.60 to 2.93), both of which are recognised antecedents of visual impairment. The most commonly diagnosed condition for those with visual impairment was hypertension, recorded for 55.6% compared to 46.7% of controls (OR 1.40, 95% CI 1.33 to 1.48).

**Table 3:** **Prevalence and odds ratios for individual physical conditions (standardised by age, gender and deprivation score)**

**Condition**	**Visual impairment Number (%)**	**No visual impairment Number (%)**	**Odds ratio (95% CI) (standardised by age, gender and deprivation)**
Multiple sclerosis (MS)	25 (0.5)	700 (0.2)	3.31 (2.43 to 4.51)
Diabetes	1,386 (25.9)	37,358 (13.1)	2.76 (2.60 to 2.93)
Epilepsy	80 (1.5)	2,850 (1.0)	2.02 (1.66 to 2.46)
Stroke or transient ischaemic attack	989 (18.5)	26,683 (9.3)	1.97 (1.84 to 2.12)
Cirrhosis/chronic liver disease/alcoholic liver disease	19 (0.4)	775 (0.3)	1.97 (1.25 to 3.13) *P* = 0.004
Peripheral vascular diseases (PVD)	455 (8.5)	14,199 (5.0)	1.74 (1.58 to 1.92)
Constipation	993 (18.6)	24,523 (8.6)	1.72 (1.59 to 1.86)
Psoriasis or eczema	84 (1.6)	2,929 (1.0)	1.67 (1.35 to 2.07)
Heart failure	505 (9.4)	14,263 (5.0)	1.64 (1.48 to 1.81)
Hearing loss	944 (17.7)	26,632 (9.3)	1.60 (1.48 to 1.73)
Chronic sinusitis	51 (0.9)	2,101 (0.7)	1.58 (1.19 to 2.09)
Chronic kidney disease	897 (16.8)	27,515 (9.6)	1.55 (1.43 to 1.68)
Bronchitis, emphysema and other chronic obstructive pulmonary diseases (COPD)	748 (14.0)	28,790 (10.1)	1.51 (1.40 to 1.63)
Inflammatory arthritis	778 (14.6)	28,097 (9.8)	1.50 (1.39 to 1.63)
Inflammatory bowel disease	66 (1.2)	2,691 (0.9)	1.49 (1.18 to 1.83) *P* = 0.01
Hypertension	2,972 (55.6)	133,429 (46.7)	1.40 (1.33 to 1.48)
Prostate disease	262 (4.9)	10,164 (3.6)	1.38 (1.22 to 1.57)
Coronary heart disease	1,542 (28.8)	58,160 (20.4)	1.39 (1.31 to 1.48)
Parkinson’s disease	75 (1.4)	2,257 (0.8)	1.34 (1.03 to 1.75)
Painful condition	1,154 (21.6)	54,120 (18.9)	1.29 (1.21 to 1.37)
Irritable bowel syndrome	243 (4.5)	11,450 (4.0)	1.28 (1.13 to 1.45)
Atrial fibrillation	599 (11.2)	19,107 (6.7)	1.26 (1.15 to 1.40)
Dyspepsia	729 (13.6)	32,848 (11.5)	1.22 (1.12 to 1.32)
Diverticular	664 (12.4)	24,053 (8.4)	1.21 (1.11 to 1.33)
Asthma (active)	348 (6.5)	18,654 (6.5)	1.20 (1.08 to 1.32)
New cancer in the last 5 years	578 (10.1)	23,638 (8.3)	1.19 (1.08 to 1.30)
Thyrotoxicosis/thyroid disorders (includes hypothyroidism)	724 (13.5)	31,757 (11.1)	1.14 (1.05 to 1.23) *P* = 0.03
Bronchiectasis	31 (0.6)	1,575 (0.6)	1.07 (0.75 to 1.57) *P* = 0.69
Migraine	16 (0.3)	1,207 (0.4)	1.02 (0.67 to 1.54) *P* = 0.74

All difference significant at *P* <0.001 except where stated. Conditions are ordered by size of odds ratio (largest to smallest).

### Mental health conditions: visual impairment versus controls

Depression had the largest prevalence for the individual mental health conditions (visually impaired 18.2% versus controls 12.0%; OR 1.53, 95% CI 1.43 to 1.65) and Table 4 highlights that those with visual impairment had significantly higher prevalence for all eight mental health conditions. The biggest difference after standardisation for age, sex and deprivation was for learning disability (visually impaired 0.5% versus controls 0.2%; OR 3.31, 95% CI 2.20 to 4.97), followed by anorexia or bulimia (visually impaired 0.5% versus controls 0.2%; OR 2.23, 95% CI 1.51 to 3.28) (although clearly the absolute prevalences of these conditions were low) and for any ‘other psychoactive substance misuse’ which is a heterogeneous grouping of problematic use of non-prescription and prescription drugs ever recorded (visually impaired 12.9% versus controls 4.8%; OR 1.79, 95% CI 1.65 to 1.95).

**Table 4:** **Prevalence and odds ratios for individual mental health conditions (standardised by age, gender and deprivation score)**

**Condition**	**Visual impairment Number (%)**	**No visual impairment Number (%)**	**Odds ratio (95% CI) (standardised by age, gender and deprivation)**
Learning disability	25 (0.5)	559 (0.2)	3.31 (2.20 to 4.97)
Anorexia or bulimia	28 (0.5)	572 (0.2)	2.23 (1.51 to 3.28)
Other psychoactive substance misuse	688 (12.9)	13,568 (4.8)	1.79 (1.65 to 1.95)
Anxiety and other neurotic, stress related and somatoform disorders	763 (14.3)	23,015 (8.1)	1.60 (1.47 to 1.73)
Depression	972 (18.2)	35,067 (12.0)	1.53 (1.43 to 1.65)
Alcohol misuse	174 (3.3)	8,565 (3.0)	1.46 (1.25 to 1.71)
Schizophrenia (and related non-organic psychosis) or bipolar disorder	75 (1.4)	2,873 (1.0)	1.42 (1.13 to 1.80) *P* = 0.003
Dementia	418 (7.2)	10,110 (3.5)	1.14 (1.02 to 2.27) *P* = 0.01

All difference significant at *P* <0.001 except where stated. Conditions are ordered by size of odds ratio (largest to smallest).

## Discussion

We found that patients aged 65 years and older with visual impairment within primary care were characterised by more medical comorbidities relative to non-visually impaired controls and that these differences were not accounted for by age, gender and social deprivation. Indeed, after standardisation those in the visual impairment group were twice as likely to have five or more physical/mental health comorbidities.

### Possible explanations and implications for clinicians and policy makers

Causality cannot be directly inferred from these cross-sectional data, but it is known that many chronic conditions have visual consequences. For example, optic neuritis occurs in 70% of MS cases [21], the ocular complications of diabetes account for the majority of blind registrations in the UK among working age adults [22] and stroke causes visual impairment in 30% of cases [23]. Other conditions, such as learning disability (more than three times more likely in the visual impairment group) are associated with a high prevalence of visual defects from childhood [24]. Notably, hypertension (55.6%), coronary heart disease (28.8%) and diabetes (25.9%) were the most prevalent conditions in the visually impaired group, and visual impairment may pose significant barriers to encouraging a healthy lifestyle in this population. Visual impairment may contribute to a compromised diet (due to difficulty in preparing food), increased difficulties engaging in exercise, and high levels of isolation and loneliness. Management of these conditions in patients with visual impairment will require active integration of clinical, social and rehabilitation services to help improve health.

Clinicians also need to be alert to depression and hearing loss which will be more common in their patients with visual impairment compared to those without, and which are likely to complicate management of other comorbid conditions they are dealing with. Visual impairment is a recognised risk factor for depression which was identified as the most prevalent mental health condition (18.2%); this is broadly consistent with previous prevalence estimates for this group (13.5% to 33% [25-27]). Comorbid hearing loss was also identified in 17.7% of people in this study, compared to only 9.3% in controls. Both depression and hearing loss are commonly underdiagnosed within primary care services [28,29] and have been related to loss of functional independence, increased risk of falls, hospitalisation and mortality [30-32]. Identifying and managing depression and hearing loss will require increased integration between services (including rehabilitation and social services) and clear referral pathways. Integration of depression screening and integrated depression treatment within eye care services is currently being evaluated [33,34]. Recommendations have also been made to integrate hearing screening tests into the eye care and rehabilitation services, which could substantially increase the numbers of people identified with hearing loss [28,35]. There is also recent evidence of Governmental commitment to tackling comorbid depression and dual sensory loss at national levels [36]. These advancements represent a move towards integrating services, but continuing integration to provide holistic care for patients will require more evidence based recommendations and commitment from the healthcare community.

Conversely, checking for and taking account of visual impairment is important for those providing care for other conditions. Clinicians need to be mindful of the increased falls risk in visual impairment when prescribing drugs for comorbid physical/mental health conditions which could also contribute to falls (for example, hypotensive and hypoglycaemic agents, bladder anticholinergics, opioid analgesics, sedative and psychotropic drugs), or in relation to safe medicines use especially when regimens are complex.

### Strengths and weaknesses

The study is a cross sectional analysis of patients in primary care using diagnostic criteria based on records within a primary care database. Recording of disease will, therefore, not be as accurate or consistent as it would be in a more formal epidemiological survey. Compared to the relatively few studies which have examined comorbidity in visual impairment [16,17], strengths of our study include the large sample size and the assessment of a much wider range of comorbid physical and mental conditions. Further, this study does not rely on patient self-report of comorbidities and takes account of confounders, such as age, gender and social deprivation. An important limitation is that visual impairment is based on GP recording of one or more Read Codes rather than formal measurement of visual function. The Read Codes used were a broad set which do not always clearly define the exact nature of the visual impairment, and visual impairment is under-recorded compared to epidemiological estimates [1]. However, our expectation is that most people with very severe impairment will have been coded. From this perspective, the finding that 1.8% of the sample was defined as having visual impairment is consistent with 0.5% of Scottish residents being formally registered as visually impaired, which is known to include only about half of those eligible for registration [37]. Registration can only be done by a consultant ophthalmologist with clear visual acuity and visual field criteria defining ‘partially-sighted’ or ‘blind’ registration. The study reported by Van Nispen [17] had clear clinical inclusion criteria for patients with visual impairment, including assessment by an ophthalmologist, but it is important to recognise that this requirement in itself is likely systematically to exclude the very frail and people who are housebound or resident in care-homes. All studies in this area are, therefore, likely to be biased in some way, and the limitations of our study are inevitable in all studies relying on routine clinical coding, even in Scottish primary care where use of electronic medical records is longstanding and coding reasonably reliable. However, these limitations have to be balanced against the ability to analyse data from very large representative samples, and we believe the findings are valid.

Additional limitations are that there may be reporting bias, where people who attend frequently are more likely to have all clinical conditions recorded, in which case people with visual impairment recorded will also be more likely to have other conditions recorded, and that we were only able to control for three confounding variables. Reporting bias is likely to be present to some extent, but we believe the impact of this is likely to be small because patients in the UK are required to register with a single practice to access National Health Service care, and their clinical record is automatically transferred when they change practice. All specialists write to the GP and, therefore, populate the primary care record. Reporting bias in cradle-to-grave record is less likely than in one where records are created anew every time patients register, although may be present for conditions where patients may significantly self-care (for example constipation) than ones where registers are more complete (for example, ischaemic heart disease). If present, reporting bias would be expected to somewhat inflate the odds ratios of having comorbidity in people with visual impairment compared to those without, but would have less influence on the estimates of comorbidity rates in the visually impaired population. With regard to other confounders, we were restricted by what data were available. Clinical records do not reliably record marital status, income, occupation or education, but do reliably record address/postcode. We, therefore, used postcode assigned socioeconomic status, gender and age because we had near complete data on them. However, this represents the trade-off between large and representative routine data analyses and more detailed epidemiological studies which are likely to be smaller and likely less representative (because the sickest and frailest will not take part). Moreover, indicators such as income, occupation or education are likely to be strongly correlated with each and, therefore, at least partly accounted for by measures of socioeconomic status such as the Carstairs score used in this study.

### Future research

The current study is a cross-sectional analysis. Cohort studies are required to enable causality of visual impairment and comorbidities to be explored further. Little is known about how visual impairment interacts with the genesis or progression of other diseases. For example, it is not clear how poor lifestyle factors (diet, exercise) which are compounded by visual impairment might contribute to hypertension and coronary heart disease. This is an important area for policy and research. Future research should seek to determine if there are cost-effective interventions which may help manage or prevent comorbidities, for example, nutritional and/or exercise interventions for elderly patients with visual impairment. Additionally, although there are incentives for GPs to record more accurate and complete registers of some comorbidities, this is not the case for visual impairment. Therefore, in accordance with recommendations made in the recently published Chief Medical Officer’s annual report [38], improvement in the quantity and quality of visual (and other sensory) impairment data would help inform future developments to local and national services for people with visual impairment.

## Conclusions

Comorbidity is common in patients aged 65 years and older with visual impairment, with high rates of multiple comorbidities in this group. Meeting patient needs and the challenges posed by visual impairment will require an integrated, multilevel approach which takes account of the clinical complexity of coexisting health conditions in this vulnerable patient group.

## Appendix 1

Read codes included in the visual impairment category

2B69. O/E -R-eye counts fingers only

2B6A. O/E-R-eye perceives light only

2B6B. O/E - R-eye completely blind

2B6C. O/E - R-eye sees hand movements

2B6P. O/E - pinhole R-eye sees hand movements

2B6Q. O/E - pinhole R-eye counts fingers only

2B6R. O/E - pinhole R-eye perceives light only

2B6S. O/E - pinhole R-eye completely blind

2B6T. O/E - R-eye visual acuity (corrected) 1/60

2B6V. O/E - R-eye visual acuity (corrected) 2/60

2B6W. O/E - R-eye visual acuity (corrected) 4/60

2B6X. O/E - R-eye visual acuity (corrected) 5/60

2B79. O/E -L-eye counts fingers only

2B7A. O/E-L-eye perceives light only

2B7B. O/E - L-eye completely blind

2B7C. O/E - L-eye sees hand movements

2B7P. O/E - pinhole L-eye sees hand movements

2B7Q. O/E - pinhole L-eye counts fingers only

2B7R. O/E - pinhole L-eye perceives light only

2B7S. O/E - pinhole L-eye completely blind

2B7T. O/E - L-eye visual acuity (corrected) 1/60

2B7V. O/E - L-eye visual acuity (corrected) 2/60

2B7W. O/E - L-eye visual acuity (corrected) 4/60

2B7X. O/E - L-eye visual acuity (corrected) 5/60

6688. Registered partially sighted

6689. Registered blind

668B. Poor visual acuity

668C. Certificate of vision impairment

F49. Blindness and low vision

F490. Blindness, both eyes

F4900 Unspecified blindness both eyes

F4901 Both eyes total visual impairment

F4902 Better eye: near total VI, Lesser eye: unspecified

F4903 Better eye: near total VI, Lesser eye: total VI

F4904 Better eye: near total VI, Lesser eye: near total VI

F4905 Better eye: profound VI, Lesser eye: unspecified

F4906 Better eye: profound VI, Lesser eye: total VI

F4907 Better eye: profound VI, Lesser eye: near total VI

F4908 Better eye: profound VI, Lesser eye: profound VI

F4909 Acquired blindness, both eyes

F490z Blindness both eyes NOS

F491. Better eye: low vision, Lesser eye: profound VI

F4910 One eye blind, one eye low vision

F4911 Better eye: severe VI, Lesser eye: blind, unspecified

F4912 Better eye: severe VI, Lesser eye: total VI

F4913 Better eye: severe VI, Lesser eye: near total VI

F4914 Better eye: severe VI, Lesser eye: profound VI

F4915 Better eye: moderate VI, Lesser eye: blind, unspecified

F4916 Better eye: moderate VI, Lesser eye: total VI

F4917 Better eye: moderate VI, Lesser eye: near total VI

F4918 Better eye: moderate VI, Lesser eye: profound VI

F491z One eye blind, one eye low vision NOS

F492. Low vision, both eyes

F4920 Low vision, both eyes unspecified

F4921 Better eye: severe VI, Lesser eye: low vision unspecified

F4922 Better eye: severe VI, Lesser eye: severe VI

F4923 Better eye: moderate VI, Lesser eye: low vision unspecified

F4924 Better eye: moderate VI, Lesser eye: severe VI

F4925 Better eye: moderate VI, Lesser eye: moderate VI

F492z Low vision, both eyes NOS

F493. Visual loss, both eyes unqualified

F494. Legal blindness USA

F495. Profound impairment, one eye

F4950 Blindness, one eye, unspecified

F4951 Lesser eye: total visual impairment, Better eye: unspecified

F4952 Lesser eye: total VI, Better eye: near normal vision

F4953 Lesser eye: total VI, Better eye: normal vision

F4954 Lesser eye: near total VI, Better eye: unspecified

F4955 Lesser eye: near total VI, Better eye: near normal vision

F4956 Lesser eye: near total VI, Better eye: normal vision

F4957 Lesser eye: profound VI, Better eye: unspecified

F4958 Lesser eye: profound VI, Better eye: near normal vision

F4959 Lesser eye: profound VI, Better eye: normal vision

F495A Acquired blindness, one eye

F495z Profound impairment one eye NOS

F496. Low vision, one eye

F4960 Low vision, one eye, unspecified

F4961 Lesser eye: severe VI, Better eye: unspecified

F4962 Lesser eye: severe VI, Better eye: near normal vision

F4963 Lesser eye: severe VI, Better eye: normal vision

F4964 Lesser eye: moderate VI, Better eye: unspecified

F4965 Lesser eye: moderate VI, Better eye: near normal vision

F4966 Lesser eye: moderate VI, Better eye: normal vision

F496z Low vision, one eye NOS

F49y. Visual loss, one eye, unqualified

F49z. Visual loss NOS

F49z0 Charles Bonnet syndrome

FyuL. [X]Visual disturbances and blindness

ZV410 [V]Problems with sight

## Additional file

Additional file 1: Table S1. Definitions of 32 physical health conditions assessed.
