# Supplementary material for: Visual impairment is associated with physical and mental comorbidities in older adults: a cross-sectional study
Source: BMC Med. 2014 Oct 17;12:181. doi: 10.1186/s12916-014-0181-7 (PMC4200167; doi:10.1186/s12916-014-0181-7)
Supplement: Additional file 1: Table S1. — Definitions of 32 physical health conditions assessed. [file 12916_2014_181_MOESM1_ESM.doc]

Additional file 1

Table S1: Definitions of 32 physical health conditions assessed

| Condition | Variable Definition |
| --- | --- |
| Coronary heart disease | Read code ever recorded |
| Chronic kidney disease | Read code ever recorded |
| Asthma (active) | Read code ever recorded AND any prescription in last year |
| Atrial fibrillation | Read code ever recorded |
| Epilepsy | Read code ever recorded AND epilepsy prescription in last year |
| New cancer in the last five years | Read code first recorded in last five years (Relevant Read Code recorded) |
| Thyrotoxicosis/thyroid disorders (includes hypothyroidism) | Read code ever recorded (Relevant Read Code recorded) |
| Diabetes | Read code ever recorded |
| Parkinson’s disease | Read code ever recorded (Relevant Read Code recorded) |
| Multiple sclerosis | Read code ever recorded (Relevant Read Code recorded) |
| Stroke or transient ischaemic attack | Read code ever recorded (Relevant Read Code recorded) |
| Visual impairment | Read code ever recorded (Relevant Read Code recorded) |
| Glaucoma | Read code ever recorded (Relevant Read Code recorded) |
| Hearing loss | Read code ever recorded (Relevant Read Code recorded) |
| Hypertension | Read code ever recorded (Relevant Read Code recorded) |
| Heart failure | Read code ever recorded |
| Peripheral vascular diseases | Read code ever recorded (Relevant Read Code recorded) |
| Chronic sinusitis | Read code ever recorded (Relevant Read Code recorded) |
| Bronchitis, emphysema and other chronic obstructive pulmonary diseases | Read code ever recorded (Relevant Read Code recorded) |
| Bronchiectasis | Read code ever recorded (Relevant Read Code recorded) |
| Crohn’s disease and ulcerative colitis | Read code ever recorded (Relevant Read Code recorded) |
| Diverticular disease of intestine | Read code ever recorded (Relevant Read Code recorded) |
| Rheumatoid arthritis, other inflammatory polyarthropathies and systematic connective tissue disorders | Read code ever recorded (Relevant Read Code recorded) |
| Hyperplasia of prostate and prostate disorders | Read code ever recorded (Relevant Read Code recorded) |
| Psoriasis or eczema | Read code ever recorded (M11% and M12%) AND ≥ four prescriptions in last year (BNF 13.4, excluding hydrocortisone, and BNF 13.5) |
| Viral hepatitis | Read code ever recorded (Relevant Read Code recorded) |
| Irritable bowel syndrome | Read code ever recorded (Relevant Read Code recorded) OR ≥4 antispasmodic prescriptions in last year (POM only, exclude kolanticon, alverine citrate and peppermint oil) |
| Cirrhosis/chronic liver disease/alcoholic liver disease | Read code ever recorded (Relevant Read Code recorded) |
| Migraine | ≥4 anti-migraine prescriptions in last year (BNF 040704%, POM only exclude migraleve) |
| Dyspepsia | ≥4 prescriptions in last year BNF 0103% excluding antacids AND NOT ≥4 NSAIDS OR ≥4 aspirin/clopidogrel |
| Constipation | ≥4 prescriptions in last year, BNF 0106% |
| Pain | ≥4 specified analgesic prescriptions in last year (opioids/>8 mg co-codamol/NSAIDS) OR ≥4 specified anti-epileptics in the absence of an epilepsy Read code in last year (gabapentin, pregabalin and carbamazepine) |
| Definitions of 8 mental health conditions assessed | |
| Condition | Variable Definition |
| Anorexia or bulimia | Read code ever recorded |
| Other psychoactive substance misuse | Read code ever recorded |
| Anxiety and other neurotic, stress related and somatoform disorders | Read code in last 12 months OR ≥4 anxiolytic/hypnotic prescriptions in last 12 months OR ≥4 10/25 mg amitriptyline in last 12 months and do not meet the criteria for ‘Pain’ |
| Depression | Read code recorded in last 12 months OR ≥4 anti-depressant prescriptions (excluding low dose tricyclics) in last 12 months |
| Alcohol misuse | Read code ever recoded |
| Schizophrenia (and related non-organic psychosis) or bipolar disorder | Read code ever recorded/recorded in last 12 months (code dependent) OR Lithium prescribed in last 168 days |
| Dementia | Read code ever recorded |
| Learning disability | Read code ever recorded |
